# Indole Derivatives Isolated from Brown Alga *Sargassum thunbergii* Inhibit Adipogenesis through AMPK Activation in 3T3-L1 Preadipocytes

**DOI:** 10.3390/md15040119

**Published:** 2017-04-12

**Authors:** Min-Cheol Kang, Yuling Ding, Eun-A Kim, Youn Kyung Choi, Thiago De Araujo, Soo-Jin Heo, Seung-Hong Lee

**Affiliations:** 1 Department of Marine Life Sciences, Jeju National University, Jeju 690-756, Korea; mkang1@bidmc.harvard.edu; 2 Division of Endocrinology, Diabetes and Metabolism, Beth Israel Deaconess Medical Center and Harvard Medical School, Boston, MA 02445, USA; 3 Department of Animal Bio and Applied Chemistry, Konkuk University, Chungju 27478, Korea; dingyuling@naver.com; 4 Jeju International Marine Science Center for Research & Education, Korea Institute of Ocean Science &Technology (KIOST), Jeju 63349, Korea; euna0718@kioat.ac.kr (E.-A.K.); choiyk@kiost.ac.kr (Y.K.C.); 5 Laboratory of Cell Signaling, Obesity and Comorbidities Research Center, University of Campinas-UNICAMP, Campinas 02134, Brazil; thiagomatosaraujo@gmail.com; 6 Division of Food Bioscience and Korea Nokyong Research Center, Konkuk University, Chungju 27478, Korea

**Keywords:** anti-obesity effect, adipocyte, *Sargassum thunbergii*, indole derivatives

## Abstract

Seaweed, a popular and abundant food ingredient mainly consumed in Asian countries, is a good source of bioactive compounds with anti-obesity effects. However, the anti-obesity effects of *Sargassum thunbergii* have not yet been established. In this study, we isolated six indole derivatives (STCs)—indole-2-carboxaldehyde (STC-1), indole-3-carboxaldehyde (STC-2), indole-4-carboxaldehyde (STC-3), indole-5-carboxaldehyde (STC-4), indole-6-carboxaldehyde (STC-5), and indole-7-carboxaldehyde (STC-6)—from *S. thunbergii* and evaluated their inhibitory effects on adipocyte differentiation in 3T3-L1 cells. We found that STC-1 and STC-5 resulted in non-toxic inhibition of the differentiation of 3T3-L1 adipocytes and thus selected these compounds for further study. STC-1 and STC-5 significantly inhibited lipid accumulation and downregulated the expression of peroxisome proliferator-activated receptor-γ (PPARγ), CCAAT/enhancer-binding protein α (C/EBPα), and sterol regulatory element-binding protein 1c (SREBP-1c) in a dose-dependent manner. The specific mechanism mediating the effects of STC-1 and STC-5 was shown to be AMP-activated protein kinase (AMPK) activation. Our results demonstrated the inhibitory effect of STC-1 and STC-5 on adipogenesis through the activation of the AMPK signal pathway. Together, these findings suggested that STC-1 and STC-5 may be effective candidates for the prevention of obesity or obesity-related diseases.

## 1. Introduction

Obesity has become a serious problem in the 21st century. Predominantly caused by the loss of control between calories ingested and calories expended [1], it is a strong risk factor for metabolic disorders including type 2 diabetes caused by insulin resistance, cardiovascular diseases, some types of cancer, and hepatic steatosis [2,3]. Adiposity is an important factor for human survival; however, an excess of white adipose tissue leads to an overload of leptin production, which causes a dysregulation in the central nervous system (CNS), exacerbating the amount of food ingested and favoring weight gain [4,5]. The preadipocyte 3T3-L1 cell line is an excellent model for the study of the inhibition of adipogenesis and various stages of obesity [6]. Consequently, many studies have utilized the 3T3-L1 cell line to search for new anti-obesity agents.

Synthetic therapeutic drugs, such as Xenical^®^ (Olistate) and Reductil^®^ (Sibutramine), have been used in the treatment of obesity. However, the reported side effects of these drugs include tension headaches, thirst, insomnia, constipation, and steatorrhea [7,8]. Consequently, many researchers have focused intensively on the identification of natural substances to treat obesity-related effects. Many anti-obesity agents from natural substances have shown promise, such as *Eucommia ulmoides*, *Lythrum salicaria* L., and *Panax japonicas*, which demonstrated a mechanism that delayed the intestinal absorption of fat by the inhibition of pancreatic lipase activity [9,10,11]. Seaweeds are known to provide an abundance of bioactive compounds with valuable biomedical and pharmaceutical potential [12]. Recently, the bioactive compounds of brown seaweeds were shown to offer potential in the management of obesity and obesity-related chronic diseases [13,14,15]. A wide range of extracts and bioactive compounds have been isolated from the brown alga *Sargassum thunbergii*, which possesses a variety of biological activities, including antioxidant activity, anti-inflammatory effects, neuroprotective effects, inhibitory effects on oxidative stress, and antibacterial effects [16,17,18,19]. However, there are no reports on the effect of *S. thunbergii* on adipocyte differentiation related to obesity.

In the present study, we isolated indole derivatives (STCs) from *S. thunbergii* and first investigated their inhibitory effect on adipocyte differentiation in 3T3-L1 cells by the measurement of lipid accumulation and the expression levels of proteins associated with adipogenesis. Moreover, we examined the mechanism to determine whether 5′-adenosine monophosphate-activated protein kinase (AMPK) activation was critical for the anti-obesity function of STCs.

## 2. Results

### 2.1. Cytotoxicity and Effect of STCs on Intracellular Lipid Accumulation in Adipocytes

**Figure 1:**
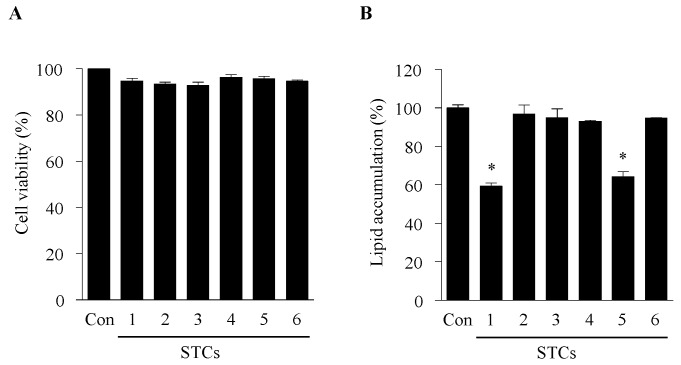
Inhibitory effect of six indole derivatives (STCs) from *S. thunbergii* on lipid accumulation in 3T3-L1 adipocytes. The effects of the compounds on the cell viability of 3T3-L1 preadipocytes treated for 48 h (**A**). Measurement of intracellular lipid accumulation was performed by Oil Red O staining (**B**). Data are expressed as the means ± SE (*n* = 3). Significant differences from the control group were identified at * *p* < 0.05.

The effect of six STCs on cell viability was measured by the 3-(4,5-dimethylthiazol-2-yl)-2,5-diphenyl tetrazolium bromide (MTT) assay. No cytotoxic effects were observed in 3T3-L1 preadipocytes at 100 µM (Figure 1A). Therefore, the test compounds were used up to this concentration for the subsequent experiments. The anti-adipogenic effects of the six STCs on the differentiation of preadipocytes into adipocytes were examined. The 3T3-L1 preadipocytes were exposed to the six STCs for 8 days (from days 0–8). Lipid accumulation was considered as a measurement of adipogenesis and was quantified at the end of the proposed differentiation period by using Oil Red O staining. Among the tested compounds, STC-1 and STC-5 exerted the highest inhibitory effect on adipogenesis (Figure 1B). Thus, we selected these compounds for further experiments.

### 2.2. Effect of STC-1 and STC-5 on Intracellular Lipid Accumulation in Adipocytes

**Figure 2:**
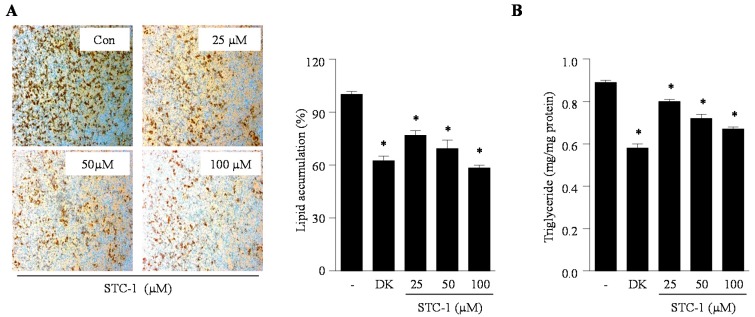
STC-1 inhibits intracellular lipid accumulation in 3T3-L1 adipocytes. Lipid accumulation was determined by measuring of Oil Red O staining (**A**) and triglyceride levels (**B**). The natural anti-adipogenic agent, dieckol (DK, 50 µM), was employed as a positive control. Data are expressed as the means ± SE (*n* = 3). Significant differences from the control group were identified at * *p* < 0.05.

**Figure 3:**
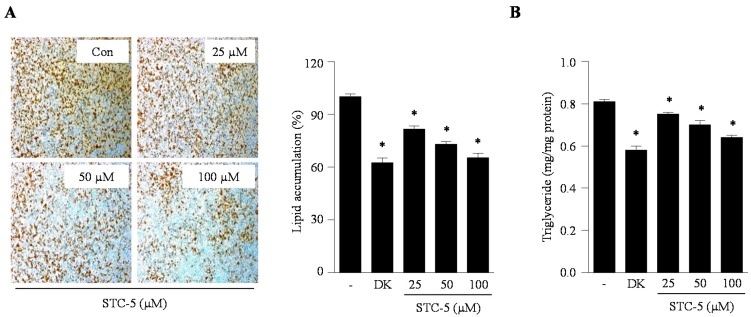
STC-5 inhibits intracellular lipid accumulation in 3T3-L1 adipocytes. Lipid accumulation was determined by measuring of Oil Red O staining (**A**) and triglyceride levels (**B**). The natural anti-adipogenic agent, dieckol (DK, 50 µM), was employed as a positive control. Data are expressed as the means ± SE (*n* = 3). Significant differences from the control group were identified at * *p* < 0.05.

Lipid accumulation, a major marker of adipogenesis, was quantified by direct Oil Red O staining and triglyceride measurements. We investigated the inhibitory effect of STC-1 and STC-5 on adipogenesis in 3T3-L1 adipocytes through the measurement of intracellular lipid accumulation and triglyceride content. As shown in Figure 2 and Figure 3, STC-1 and STC-5 significantly reduced the intracellular lipid accumulation in differentiated adipocytes in a dose-dependent manner (Figure 2A and Figure 3A). Furthermore, STC-1 and STC-5 significantly decreased the intracellular triglyceride levels compared with untreated control cells at all the tested concentrations of STC-1 and STC-5 (Figure 2B and Figure 3B). In particular, treatment with 100 µM STC-1 and STC-5 showed a similar inhibitory effect on intracellular lipid accumulation as that of the known natural anti-adipogenic agent dieckol. These results indicated that STC-1 and STC-5 effectively blocked the formation of mature 3T3-L1 adipocytes.

### 2.3. Effect of STC-1 and STC-5 on the Expression of Adipogenic-Specific Proteins

**Figure 4:**
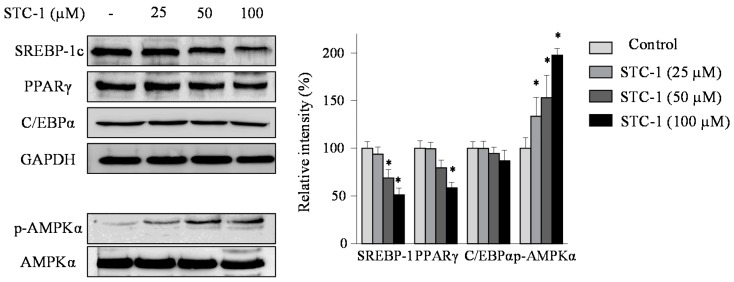
The effect of treatment with STC-1 on the expression of adipogenic-specific and AMP-activated protein kinase (AMPK) phosphorylation in 3T3-L1 adipocytes. The 3T3-L1 preadipocytes were incubated in differentiation medium without or with the indicated concentrations of STC-1 for 8 days (from day 0 to day 8). The expression of sterol regulatory element-binding protein 1c (SREBP-1c), peroxisome proliferator-activated receptor-γ (PPARγ), CCAAT/enhancer-binding protein α (C/EBPα) and AMPK was assessed by Western blotting as described in the Materials and Methods. Data are expressed as the means ± SE (*n* = 3). Significant differences from the control group were identified at * *p* < 0.05.

**Figure 5:**
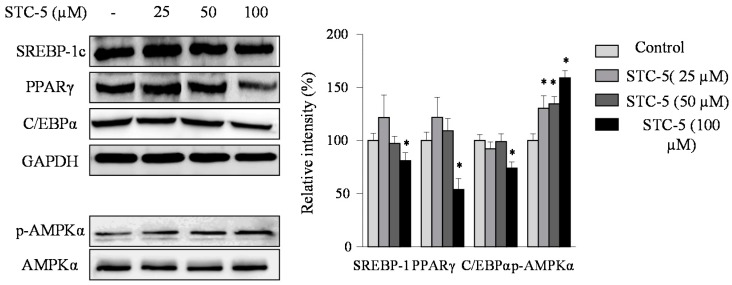
The effect of treatment with STC-5 on the expression of adipogenic-specific and AMPK phosphorylation in 3T3-L1 adipocytes. The 3T3-L1 preadipocytes were incubated in differentiation medium without or with the indicated concentrations of STC-5 for 8 days (from day 0 to day 8). The expression of SREBP-1c, PPARγ, C/EBPα and AMPK was assessed by Western blotting as described in the Materials and Methods. Data are expressed as the means ± SE (*n* = 3). Significant differences from the control group were identified at * *p* < 0.05.

To elucidate the molecular mechanisms by which STC-1 and STC-5 inhibits adipogenesis, its effect on the protein expression of the adipogenic transcription factors sterol regulatory element-binding protein 1c (SREBP-1c), peroxisome proliferator-activated receptor-γ (PPARγ), and CCAAT/enhancer-binding protein α (C/EBPα) was investigated using Western blot analysis (Figure 4 and Figure 5). As expected, the results showed that the presence of STC-1 and STC-5 significantly reduced the protein expression levels of these three transcription factors. These data suggest that STC-1 and STC-5 result in the down-regulated expression of adipogenesis transcription factors, with the subsequent inhibition of adipocyte differentiation.

### 2.4. Effect of STC-1 and STC-5 on AMPK Activation

To determine whether the inhibition of adipocyte differentiation by STC-1 and STC-5 was mediated by AMPK activation, we examined the protein levels of phosphorylated AMPK using Western blot analysis (Figure 4 and Figure 5). STC-1 and STC-5 enhanced the phosphorylation of AMPK in a dose-dependent manner. These results suggested that STC-1 and STC-5 inhibited adipogenesis by AMPK activation.

## 3. Discussion

Obesity is an increasing health problem in the 21st century and is characterized by the excessive accumulation of body fat. Excessive fat accumulation can induce metabolic syndromes, including heart disease, type 2 diabetes, high blood pressure, and stroke [20,21]. The differentiation of preadipocytes into mature adipocytes and the accumulation of fat play key roles in the pathogenesis of obesity [22]. Recently, many seaweed-derived bioactive compounds have been evaluated for their ability to inhibit adipogenesis [13,14,15]. Owing to their anti-obesity effect, these bioactive compounds may be excellent sources in the development of biomedical agents against obesity.

Many reports on indole derivatives isolated from natural sources, including marine algae, have indicated a variety of biological activities [23,24]. However, there is currently a lack of evidence on the effects of indole derivatives on adipogenesis related to obesity. Therefore, in the present study, we isolated six indole derivatives (STCs) from *S. thunbergii* and first investigated their potential inhibitory effect on adipocyte differentiation in 3T3-L1 cells. Then, we selected STC-1 and STC-5 among six STCs owing to their higher inhibitory effect on differentiation in 3T3-L1 cells. Thereafter, the anti-adipogenic effects of STC-1 and STC-5 by measurement of lipid accumulation and the expression of several adipogenesis-related proteins were investigated in differentiated 3T3-L1 cells. STC-1 and STC-5 treatment dose-dependently reduced the triglyceride levels in addition with the absorbance values of Oil Red O solution leached into the cytoplasm of treated cells, which suggested that the mechanism of STC-1 and STC-5 proceeded by the inhibition of adipogenesis through a reduction in lipid accumulation. The present study is the first to demonstrate the efficacy of STC-1 and STC-5 in this way. Accordingly, the evaluation of the mechanism action of STC-1 and STC-5 in 3T3-L1 adipocytes would be of great interest.

Adipocytes play a vital role in lipid homeostasis and energy balance; adipose-specific proteins have an important role in maintaining the size and the number of adipocytes during adipogenesis and lipogenesis [25]. Adipogenic-specific proteins are markers of fat accumulation and should be controlled to attenuate fat accumulation following differentiation in preadipocyte [26]. Adipogenesis is a process modulated by adipogenic-specific proteins during preadipocyte differentiation, including SREBP-1c, PPARγ and C/EBPα. Previous studies have reported that the levels of adipogenic-specific proteins were significantly increased in mouse models of obesity [27,28]. Therefore, the overexpression of these adipogenic specific proteins can accelerate adipogenesis. To evaluate whether STC-1 and STC-5 possessed the ability to inhibit adipocyte differentiation through SREBP-1c, PPARγ and C/EBPα, the expression of these proteins was measured using Western blot analysis. Compared with control adipocytes, the expression of SREBP-1c, PPARγ and C/EBPα was downregulated in the presence of STC-1 and STC-5, which supported the possibility that STC-1 and STC-5 may inhibit lipid accumulation by blocking the expression of these adipogenesis-specific proteins.

The 5′-adenosine monophosphate-activated protein kinase (AMPK) system plays a key role in the regulation of energy balance and glucose metabolism in the human body. It has been previously reported that AMPK has an important role in human energy metabolism and that the stimulation of AMPK induced changes in adiposity which were associated with the prevention of obesity [29,30]. Recently, it was reported that bioactive compounds from seaweed, such as dieckol and dioxinodehydroeckol, were found to inhibit adipogenesis in 3T3-L1 cells via an unusual pathway, the activation of AMPK signaling [14,31]. In this study, we used dieckol as a positive control, which caused a reduction in intracellular lipid accumulation. Therefore, in order to elucidate the molecular mechanism by which STC-1 and STC-5 inhibited adipogenesis through AMPK activation, the protein level of phosphorylated AMPK was measured. Treatment with STC-1 and STC-5 induced the phosphorylation of AMPK. Therefore, the results of this study suggested that STC-1 and STC-5 inhibited adipogenesis through the upregulation of AMPK activation.

The current study possessed certain limitations; there are still important issues that remain unresolved, including the anti-obesity effects of STC-1, STC-5, or the compound-rich extract in animal models and human studies. However, our findings presented herein suggested that STC-1 and STC-5 may be promising novel compounds for the treatment of obesity and related diseases.

## 4. Materials and Methods

### 4.1. Materials

The brown alga *Sargassum thunbergii* was collected from the coast of Jeju Island, Korea. All collected alga samples were washed three times with tap water to remove salt, sand, and epiphytes attached to the surface, followed by careful rinsing with fresh water and maintenance in a medical refrigerator at −20 °C. Next, the frozen samples were lyophilized and homogenized with a grinder prior to extraction.

Dulbecco’s modified Eagle’s medium (DMEM), fetal bovine serum (FBS), bovine serum (BS), Phosphate-buffered saline (pH 7.4; PBS) and penicillin–streptomycin (PS) were purchased from Gibco BRL (Grand Island, NY, USA). Antibodies against peroxisome proliferator activated receptor gamma (PPARγ) and CCAAT/enhancer binding protein (C/EBPα) were purchased from Cell Signaling Technology (Bedford, MA, USA). Antibodies against sterol regulatory element binding protein 1c (SREBP-1c) and glyceraldehyde 3-phosphate dehydrogenase (GAPDH) were from Santa Cruz Biotechnology (Santa Cruz, CA, USA). The 3-isobutyl-1-methylxanthine (IBMX), dexamethasone, insulin, and 3-(4,5-dimethylthiazol-2-yl)-2,5-diphenyl tetrazolium bromide (MTT) were obtained from Sigma Chemical Co. (St. Louis, MO, USA). The 3T3-L1 preadipocytes were obtained from American Type Culture Collection (Rockville, MD, USA). All chemicals and reagents used were of analytical grade and were obtained from commercial sources.

### 4.2. Extraction and Isolation

**Figure 6:**
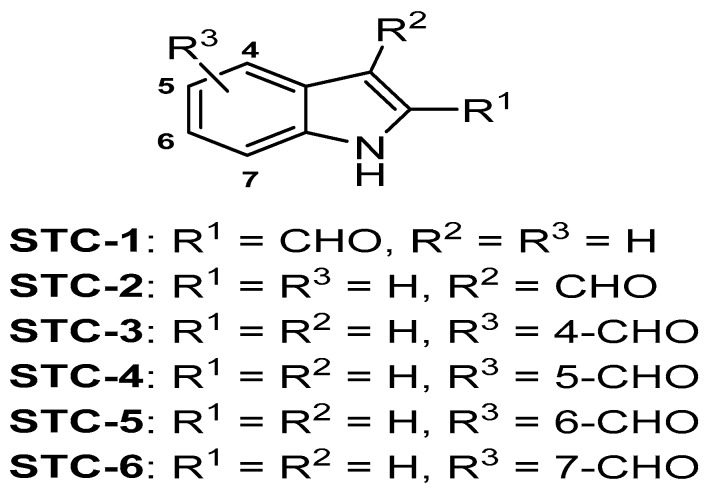

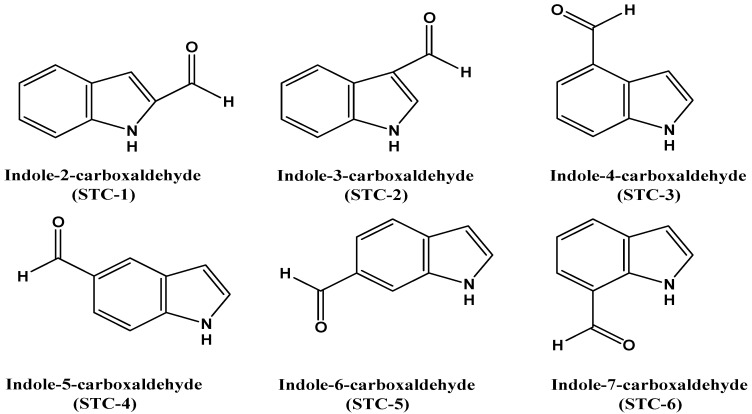
Chemical structure of indole derivatives isolated from *Sargassum thunbergii*.

Dried *S. thunbergii* powder was extracted three times with 80% methanol and filtered. The filtrate was evaporated at 40 °C to obtain the methanol extract, which was suspended in distilled water and partitioned using chloroform. The chloroform fraction was fractionated by silica column chromatography with stepwise elution of chloroform–methanol mixture (30:1→1:1) to separate active fractions in chloroform extract. A combined active fraction was further subjected to a Sephadex LH-20 column saturated with 100% methanol, and then purified by reversed phase high performance liquid chromatography (HPLC) using a Waters HPLC system (Alliance 2690; Waters Corp., Milford, MA, USA) equipped with a Waters 996 photodiode array detector and C18 column (J’sphere ODS-H80, 250 × 4.6 mm, 4 μm; YMC Co., Kyoto, Japan) by stepwise elution with methanol–water gradient (ultraviolet absorbance detection wavelength, 296 nm; flow rate, 1 mL/min). At long last, the purified compounds were identified by comparing their ^1^H- and ^13^C-NMR data with literature [32]. Figure 6 indicate the chemical structures of indole derivatives. The compound was dissolved in dimethylsulfoxide (DMSO) and employed in experiments in which the final concentration of DMSO in culture medium was adjusted to <0.01%.

### 4.3. Cell Culture and Differentiation

The 3T3-L1 preadipocytes were cultured in DMEM containing 1% PS and 10% BS at 37 °C under a 5% CO_2_ atmosphere. The differentiation induction was performed after 2-day post preadipocytes confluence (designated Day 0). The cells were cultured in MDI (IBMX, dexamethasone and insulin) differentiation medium (DMEM containing 1% PS, 10% FBS, 0.5 mM IBMX, 0.25 μM dexamethasone and 5 μg/mL insulin) for 2 days. The cells were then cultivated for another 2 days in DMEM containing 1% PS, 10% FBS and 5 μg/mL insulin. Thenceforth, the cells were maintained in post differentiation medium (DMEM containing 1% PS and 10% FBS), with replacement of the medium every 2 days. To examine the effects of test samples on the differentiation of preadipocytes to adipocytes, the cells were cultured with MDI in the presence of test samples. After 8 days (Day 8), adipogenic markers were used to confirm the differentiation and also the appearance of lipid droplets. Dieckol isolated from Ecklonia cava was used as a positive control because of its excellent anti-adipogenic effect [14].

### 4.4. Cytotoxic Assessment Using MTT Assay

The 3T3-L1 preadipocytes were seeded onto 24-well plates. The cells were treated with designated samples. The cells were then incubated for an additional 48 h at 37 °C. MTT solution (50 μL; 2 mg/mL in PBS) was added to the medium and incubated for 4 h. The purple formazan crystals were dissolved in 150 μL DMSO and the absorbance was read at 540 nm on the ELISA plate reader.

### 4.5. Determination of Lipid Accumulation by Oil Red O Staining

Cells were seeded in 6-well plates and adipocyte differentiation was induced for 8 days as described in the cell culture and adipocyte differentiation section. On day 8, the cells were stained with Oil Red O (an indicator of cell lipid content) according to a previously described method, with slight modifications [33]. In brief, cells were washed with phosphate-buffered saline (PBS), fixed with 10% buffered formalin, and stained with Oil Red O solution (0.7 g in 200 mL of isopropanol) for 1 h at room temperature. Thereafter, the Oil Red O staining solution was removed and the plates were rinsed with distilled water and dried. Images of the stained lipid droplets were taken under a light microscope (Olympus D970; Olympus Optical Co., Tokyo, Japan). Finally, the dye retained in the cells was eluted with isopropanol and quantified by measuring the optical absorbance at 520 nm.

### 4.6. Measurement of Triglyceride Content

Cellular triglyceride contents were determined using a commercial triglyceride colorimetric assay kit (Cayman Chemical, Ann Arbor, MI, USA) according to the manufacturer’s instructions. The cells were washed twice with PBS, scraped into a homogenizing solution, and sonicated to homogenize the cell suspension. The cell lysate was collected and centrifuged at 3000× *g* for 5 min at 4 °C. The supernatants were analyzed for the content of triglycerides and proteins. Triglycerides were normalized with the respective protein concentration, employing bovine serum albumin as the calibration standard. Results are represented as the amount of triglyceride in milligram to an equivalent of cellular proteins (in milligrams).

### 4.7. Western Blot Analysis

Cell lysates were prepared using lysis buffer containing 20 mM Tris, 10 mM Na_4_P_2_O_7_, 5 mM ethylenediaminetetraacetic acid, 100 mM NaF, 2 mM Na_3_VO_4_, 10 mg/mL aprotinin, 1% NP-40, 1 mM phenylmethylsulfonyl fluoride, and 10 mg/mL leupeptin. Cellular debris was removed by centrifugation at 10,000× *g* under 0 °C for 15 min. The protein concentrations were analyzed using the BCA Protein Assay Kit. The protein levels were normalized to 50 μg for the electrophoresis on a sodium dodecyl sulfate polyacrylamide gel (SDS-PAGE). The separated protein bands were transferred onto a nitrocellulose membrane. The nonspecific binding sites were blocked with 5% non-fat dry milk in TBST (25 mM Tris-HCl, 2.65 mM KCl, 137 mM NaCl, 0.05%, Tween 20, pH 7.4). The primary antibodies were introduced at a 1:1000 dilution, following overnight incubation at 4 °C. The membranes were gently rinsed using TBST and incubated with the respective secondary antibodies at a 1:3000 dilution. An ECL Western blotting detection kit was used to develop the signals by exposing on X-ray films.

### 4.8. Statistical Analysis

Data were analyzed using the statistical package for the social science (SPSS) package for Windows (Version 8). Values were expressed as means ± standard error (SE). A *p*-value of less than 0.05 was considered significant.

## 5. Conclusions

In conclusion, we have reported the first demonstration that STC-1 and STC-5 were able to inhibit lipid accumulation and adipogenesis in adipocytes through modulation of signaling pathways, which involved the inhibition of SREBP-1c, PPARγ and C/EBPα and the activation of AMPK. These results suggest that STC-1 and STC-5 have the potential to be developed as therapeutic agents for obesity.
